# Effect of Vildagliptin on Hepatic Steatosis

**DOI:** 10.1210/jc.2014-3794

**Published:** 2015-01-09

**Authors:** Mavin Macauley, Kieren G. Hollingsworth, Fiona E. Smith, Peter E. Thelwall, Ahmad Al-Mrabeh, Anja Schweizer, James E. Foley, Roy Taylor

**Affiliations:** Newcastle Magnetic Resonance Centre (M.M., K.G.H., F.E.S., P.E.T., A.A.-M., R.T.), Institute of Cellular Medicine, Newcastle University, Newcastle upon Tyne NE4 5PL, United Kingdom; Novartis Pharma AG (A.S.), CH-4056 Basel, Switzerland; and Novartis Pharmaceutical (J.E.F.), East Hanover, New Jersey 07936

## Abstract

**Context:**

Although dipeptidyl-peptidase-4 inhibitors exert their major action via an incretin mechanism, a favorable effect of vildagliptin on lipid metabolism remains unexplained.

**Objective:**

The objective was to examine hepatic triglyceride levels and insulin sensitivity on vildagliptin.

**Design:**

This was a 6-month, randomized, double-blind, placebo-controlled trial.

**Setting:**

This was an outpatient study at a university clinical research center.

**Patients:**

Individuals with type 2 diabetes (n = 44) and glycated hemoglobin ≤7.6% on stable metformin therapy were included.

**Intervention:**

Intervention was vildagliptin 50 mg twice a day or placebo over 6 months.

**Main Outcome Measures:**

Main outcome measures were hepatic triglyceride levels and insulin sensitivity.

**Results:**

Mean fasting liver triglyceride content decreased by 27% with vildagliptin, from 7.3 ± 1.0% (baseline) to 5.3 ± 0.9% (endpoint). There was no change in the placebo group. The between-group difference in change from baseline was significant (*P* = .013). Mean fasting plasma glucose concentration decreased over the study period with vildagliptin vs placebo by −1.0 mmol/L (*P* = .018), and there was a positive correlation between these decrements and liver triglyceride in the vildagliptin group at 3 months (r = 0.47; *P* = .02) and 6 months (r = 0.44; *P* = .03). Plasma alanine aminotransferase fell from 27.2 ± 2.8 to 20.3 ± 1.4 IU/L in the vildagliptin group (*P* = .0007), and there was a correlation between the decrements in alanine aminotransferase and liver triglyceride (r = 0.83; *P* < .0001). Insulin sensitivity during the euglycemic clamp was similar in each group at baseline (3.24 ± 0.30 vs 3.19 ± 0.38 mg/kg/min) and did not change (adjusted mean change of 0.26 ± 0.22 vs 0.32 ± 0.22 mg/kg/min; *P* = .86). Mean body weight decreased by 1.6 ± 0.5 vs 0.4 ± 0.5 kg in the vildagliptin and placebo groups, respectively (*P* = .08).

**Conclusions:**

This study demonstrates that the dipeptidyl-peptidase-4 inhibitor vildagliptin brings about a clinically significant decrease in hepatic triglyceride levels during 6 months of therapy unrelated to change in body weight. There was no change in peripheral insulin sensitivity.

Following identification of the therapeutic effect of glucagon-like peptide-1 (GLP-1), the dipeptidyl-peptidase-4 (DPP-4) inhibitors were developed specifically to delay its rapid degradation in plasma, and hence to enhance the incretin effect in type 2 diabetes (1–3). Vildagliptin achieves prolong and almost complete DPP-4 inhibition, resulting in the extension of meal induced increases in GLP-1 and gastric inhibitory peptide over 24 hours. GLP-1 and gastric inhibitory peptide increase the sensitivity of the α- and β-cells to glucose, which is accepted as their major mechanism of action (4, 5).

However, vildagliptin brings about changes that would not be predicted from its actions in the pancreas. It decreases postprandial triglyceride levels and decreases lipolysis as assessed in vivo by palmitate dilution more than can be accounted for by change in plasma insulin concentration (6, 7). This could result in a decrease in liver triglyceride concentration. Vildagliptin has also been shown to increase glucose utilization, as assessed during a two-step hyperinsulinemic euglycemic clamp at the high insulin dose (80 mU), and this could potentially be secondary to a reduction in liver fat (1, 2, 8). Whether hepatic lipid metabolism is specifically affected has not been examined, and there is no information on any modulation of liver triglyceride concentration.

The present randomized, placebo-controlled study was designed to examine the possible effects of vildagliptin on hepatic steatosis and insulin sensitivity. To minimize any indirect metabolic effects due to a large change in ambient plasma glucose levels, people with type 2 diabetes well controlled on metformin alone were studied.

## Patients and Methods

### Study protocol

A single-center, randomized, double-blind, placebo-controlled, parallel-group study was conducted. Forty-four patients with type 2 diabetes and glycated hemoglobin (HbA_1c_) ≤ 7.6%, who were treated with metformin, were randomized equally to the DPP-4 inhibitor vildagliptin (50 mg twice a day) and placebo. Two patients from the vildagliptin-treated group (one with multiple myeloma, and the other with atrial fibrillation related to chest infection) and three patients from the placebo group (one with marked deterioration in glycemic control, another with metastatic prostate cancer, and a third who withdrew consent) were withdrawn. None of these events were thought to be drug related.

Each participant attended one screening visit (wk −4; ie, 4 weeks before baseline assessments) for assessment of inclusion/exclusion criteria. Measurement of liver triglyceride and peripheral and hepatic insulin sensitivity and anthropometric tests were carried out at the Magnetic Resonance Centre on three occasions, each separated by at least 3 days. Randomization to vildagliptin or placebo was then carried out. Each person attended for eight additional visits over the 6-month period of treatment with vildagliptin 50 mg twice a day or placebo. Thereafter, measurements of liver triglyceride and peripheral insulin sensitivity and anthropometric tests were repeated. To permit judgment on the metabolic significance of any changes in liver triglyceride levels, a group of individuals with normal glucose tolerance defined by World Health Organization criteria were matched for age, weight, and sex with the randomized patients with type 2 diabetes and studied (n = 14).

The study was approved by the Newcastle and North Tyneside 2 Research Ethics Committee and was registered on the database of clinical trials (ClinicalTrials.gov, ID no. NCT01356381).

### Subjects

Subjects with type 2 diabetes (28 males and 16 females; HbA_1c_, 6.4 ± 0.1%); with a mean duration of diabetes of 5.7 ± 0.7 years were studied. All were taking metformin and no other oral hypoglycemic agent. None were taking insulin. A group of normal glucose-tolerant subjects with no first-degree relative with diabetes, matched for age, weight, and body mass index, were recruited as controls to provide comparative data. Normal glucose tolerance was demonstrated in all control subjects by a 75-g oral glucose tolerance test (mean fasting plasma glucose, 5.3 mmol/L; 2-h plasma glucose, 5.5 mmol/L). The characteristics of both groups are shown in Table 1. Informed written consent was obtained from all volunteers. All subjects underwent a 10-hour overnight fast and were advised to abstain from vigorous exercise and smoking for 3 days before test days.

**Table 1.** Characteristics of Study Groups

	Vildagliptin Group	Placebo Group	Type 2 Diabetes Group	Controls
n	22	22	44	14
Age, y	65.2 ± 0.7	58.9 ± 1.6	61.4 ± 1.0	59.0 ± 2.2
Weight, kg	83.0 ± 3.2	91.8 ± 2.4	87.2 ± 2.0	86.7 ± 3.7
BMI, kg/m^2^	29.4 ± 0.8	31.1 ± 0.6	30.3 ± 0.5	29.6 ± 1.0
Body fat, %	31.5 ± 1.6	30.7 ± 1.8	31.1 ± 1.2	27.9 ± 2.6
WHR	0.95 ± 0.01	0.95 ± 0.01	0.95 ± 0.01	0.90 ± 0.02^a^
FPG, mmol/L	7.7 ± 0.2	7.8 ± 0.3	7.9 ± 0.2	5.1 ± 0.1^b^
FPI, mU/L	13.0 ± 1.8	14.8 ± 1.6	13.5 ± 1.2	7.9 ± 0.9^b^
HOMA-IR, μU/mol/L^3^	4.4 ± 0.6	5.3 ± 0.7	4.7 ± 0.4	1.9 ± 0.2^b^
HbA_1c_, %	6.6 ± 0.1	6.5 ± 0.1	6.4 ± 0.1	-

Abbreviations: BMI, body mass index; WHR, waist/hip ratio; FPG, fasting plasma glucose; FPI, fasting plasma insulin; HOMA-IR, homeostasis model of assessment for insulin resistance. Data are shown as mean ± SEM.

a *P* ≤ .05; and

b *P* ≤ .01.

### Liver triglyceride quantitation

A Philips 3.0 T Achieva scanner with a six-channel cardiac coil (Philips Healthcare) was used to measure liver triglyceride content. Data were acquired using a three-point Dixon method (9) with three gradient-echo scans acquired with adjacent out-of-phase and in-phase echoes during a 17-second breath hold (repetition time/echo time/averages/flip angle = 50 ms/3.45, 4.60, 5.75 ms/1/5°). A matrix size of 160 × 109 and with a field view of 400–480 mm according to volunteer size was used. The fat and water contributions of the magnetic resonance imaging signal were separated using an in-house program written in MATLAB, with the triglyceride content in the images expressed as a percentage of the total signal from fat and water in each pixel. The interscan Bland-Altman repeatability coefficient has previously been reported as 0.5% (10). The polygon tool in the imaging software Image J (11) was used to define regions of interest within the homogenous liver parenchyma on five separate slices of each scan, clearly avoiding contamination of data from blood vessels, the gall bladder, or any peripheral tissue so that regions of interest solely represented intrahepatic triglyceride. The five slice measurements were then averaged.

### Euglycemic hyperinsulinemic clamp and endogenous glucose production

On the patient's arrival at the study center, an 18-gauge cannula was inserted into a wrist or hand vein. This hand was placed in a hand-warming device and heated to 55°C to achieve arterialization of the venous blood samples. A second cannula was inserted into the antecubital vein of the contralateral arm for infusion of [6′6′-^2^H] glucose (98% enriched; Cambridge Isotope Laboratories). Basal hepatic glucose production (HGP) was calculated during the last 30 minutes of the basal period (−180 to 0 min), and clamp HGP was calculated during the final 30 minutes of the clamp period (12). Plasma glucose was clamped at 5.5 mmol with an insulin infusion rate of 40 mU m^−2^min^−1^, and whole-body insulin sensitivity was determined during the last 30 minutes of the 180-minute clamp (13). To prevent marked fluctuations in plasma [6′6′-^2^H] glucose atom percentage excess, glucose clamping was carried out using 10% dextrose enriched with the isotope as previously reported (10). Whole-body glucose and lipid oxidation was measured before and between 120 and 150 minutes of the clamp using a Quark RMR indirect calorimeter (Cosmed). The equations upon which the calculations are based are not made available by the manufacturer and, although in widespread use, may be subject to error. Protein oxidation was calculated using previously validated assumptions and was not measured (14). It was assumed not to change during the 6-month study.

### Body composition and anthropometry

A bioelectrical impedance device (Bodystat 1500; Bodystat Ltd) was used to determine percentage body fat. The repeatability of the Bodystat 1500 machine was assessed for a single volunteer on 10 different occasions. The intraindividual coefficient of variation was 6.0%. Waist and hip circumferences were measured with a nondistensible tape measure with subjects in a relaxed standing position. The waist was defined as the midpoint between the lower edge of the rib cage superiorly and the anterior superior iliac spine inferiorly. The hip circumference was taken to be at the level of the greater trochanter. The waist and hip measurements were expressed as a ratio.

### Metabolite and hormone assay

Plasma glucose concentration was measured with a Yellow Springs glucose analyzer (YSI Inc). HbA_1c_ was measured by HPLC (Bio-Rad). Plasma insulin was measured with Dako Insulin ELISA (DAKO) using a spectrophotometric analyzer. Glucagon concentration was measured with a Millipore Glucagon RIA Kit (Millipore Corporation).

The ^2^H atom percentage excess in plasma glucose was determined using a Thermo “Voyager” single quadruple mass spectrometer with Thermo “Trace” gas chromatograph (Thermo Scientific). Plasma triglyceride was measured with the Triglyceride GPO-PAP spectrophotometric assay (Roche Diagnostics), using Roche/Hitachi Modular Analyzer. Nonesterified fatty acid (NEFA) was measured with an enzymatic colorimetric method assay using the Wako NEFA-HR (2) reagent (Wako Chemical). High-density lipoprotein (HDL) cholesterol was measured by Roche WAKO Direct Homogenous assay. Very low-density lipoprotein cholesterol was calculated from the total HDL cholesterol measurements.

### Data analysis

Data are expressed as mean ± SEM. The adjusted mean changes from baseline to endpoint (with the last observation carried forward for data obtained at or after the mo 3 visit) were compared between treatments using analysis of covariance.

Student's two-tailed paired *t* test or the Mann-Whitney test was used to compare within-group and between-group changes at different time points. Pearson correlation was used to assess the relationship between variables. The safety data were summarized descriptively by treatment.

## Results

### Intrahepatic triglyceride

**Figure 1.**
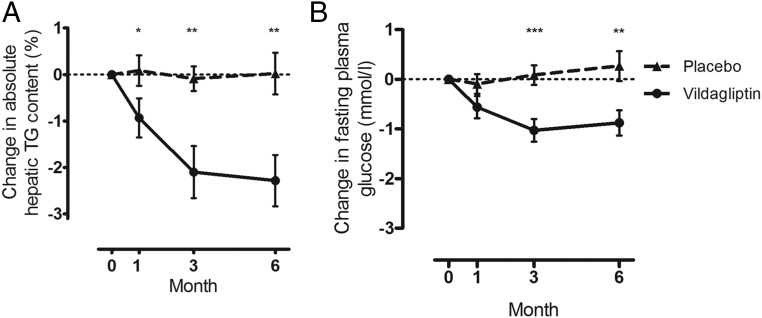
The effect of 6 months of vildagliptin on change in: A, hepatic triglyceride (TG) content (expressed as absolute change in percentage hepatic fat, not relative change); and B, fasting plasma glucose in the vildagliptin-treated and placebo groups, respectively. *, *P* < .05; **, *P* < .005; ***, *P* < .0005.

Mean fasting liver triglyceride decreased during vildagliptin treatment from 7.3 ± 1.0% at baseline to 5.3 ± 0.9% at endpoint (*P* = .001). There was no change in the placebo group (5.4 ± 0.7% to 5.4 ± 1.0%; *P* = .48). The between-group difference in change from baseline was significant (*P* = .013), representing a clinically relevant improvement in liver triglyceride. The time course of change is shown in Figure 1A. Within the vildagliptin group, intrahepatic triglyceride fell from baseline by 12% at 1 month (*P* = .04), 29% at 3 months (*P* = .001), and 27% at 6 months (*P* = .0006).

At baseline, intrahepatic triglyceride was higher in the whole type 2 diabetes group compared with the normal control group (6.7 ± 0.7 vs 3.4 ± 0.8%; *P* = .0008).

### Plasma alanine aminotransferase (ALT)

**Figure 2.**
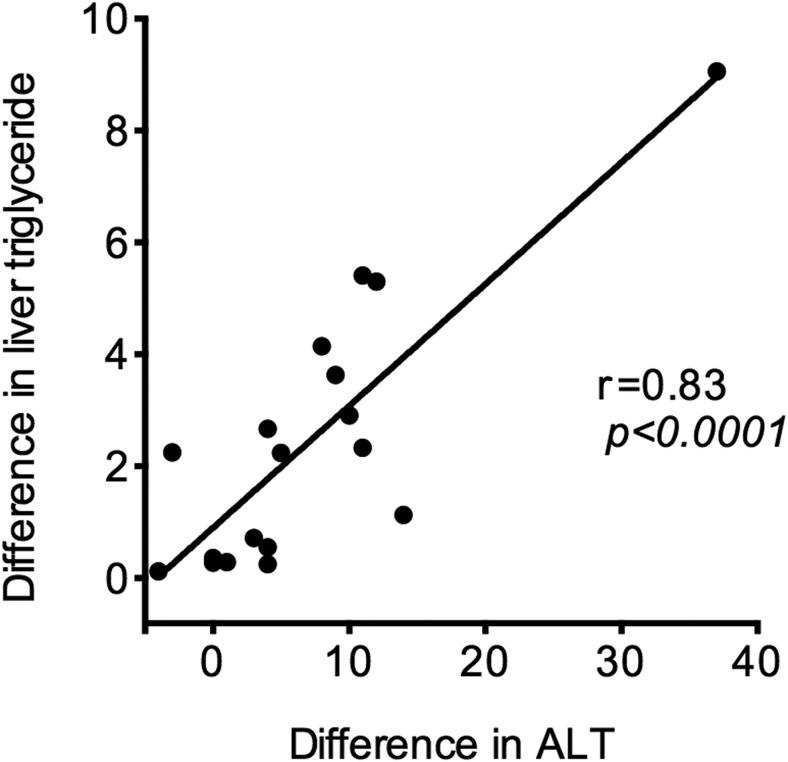
Relationship between change in liver triglyceride concentration and change in plasma ALT. The correlation remained similarly significant if the outlying point was excluded (ie, r = 0.64; *P* = .003).

Mean plasma ALT fell from 27.2 ± 2.8 to 20.3 ± 1.4 IU/L in the vildagliptin group (*P* = .0007) and did not change in the placebo group (29.6 ± 3.0 to 29.6 ± 3.7 IU/L; *P* = .44). There was a positive correlation between the fall in ALT and the fall in liver fat in the vildagliptin group (r = 0.83; *P* < .0001) (Figure 2). The correlation between these remained significant if the outlier was excluded (r = 0.64; *P* = .003).

### Whole-body insulin sensitivity assessed by euglycemic (5.5 mmol/L) hyperinsulinemic (40 mU insulin min^−2^min^−1^) clamp

Baseline mean glucose disposal rates were 3.24 ± 0.30 mg^−1^kg^−1^min^−1^ in the vildagliptin group and 3.19 ± 0.38 mg^−1^kg^−1^min^−1^ in the placebo group. Glucose disposal rates did not change meaningfully in either group from baseline to endpoint (3.24 ± 0.30 to 3.50 ± 0.31 and 3.19 ± 0.38 to 3.52 ± 0.40 mg^−1^kg^−1^min^−1^, respectively). There was no between-group difference in the change from baseline (−0.06 mg^−1^kg^−1^min^−1^; *P* = .86). When glucose disposal was expressed as a ratio to the clamp plasma insulin at steady state (M/I, calculated as glucose disposal (M) divided by steady state plasma insulin concentration), insulin sensitivity did not change at 6 months in the vildagliptin (0.0065 ± 0.0006 to 0.0071 ± 0.0006 mL/kg/min/pm) and placebo (0.0074 ± 0.0013 to 0.0078 ± 0.0013 mL/kg/min/pm) groups. There was no between-group difference in the change from baseline (0.0001 mL/kg/min/pm; *P* = .89). Steady-state concentrations of plasma glucose, insulin, and 6′6′ diduterated glucose were achieved at baseline and 6 months for the vildagliptin and placebo groups (Supplemental Figures 1 and 2).

### Hepatic glucose production

Fasting HGP at baseline was the same in the vildagliptin and placebo groups (1.95 ± 0.10 vs 1.95 ± 0.07 mg/kg/min).

Percentage suppression of hepatic glucose production during the hyperinsulinemic euglycemic clamp at baseline and endpoint was similar in the vildagliptin (42.2 ± 0.07 vs 42.8 ± 0.05%; *P* = .89) and placebo (54.7 ± 0.06 vs 48.4 ± 0.07%; *P* = .42) groups.

### Plasma HbA_1c_ and plasma glucose

Mean HbA_1c_ changed by −0.5 ± 0.1% (*P* < .0001) from a baseline of 6.5 ± 0.1% in the vildagliptin group, whereas a small numerical increase (0.2 ± 0.1% from a baseline of 6.4 ± 0.1%; *P* = .14) was seen in the placebo group, resulting in a significant between-group difference of −0.7 ± 0.1% (*P* < .001). In the vildagliptin group, most of the HbA_1c_ decrease was seen in the first 3 months (6.1 ± 0.1% at 3 mo and 6.0 ± 0.1% at 6 mo, respectively), whereas in the placebo group there was little change over the entire study period (6.4 ± 0.1% at 3 mo and 6.5 ± 0.2% at 6 mo, respectively).

Mean fasting plasma glucose changed by −0.9 ± 0.3 mmol/L (*P* = .001) in the vildagliptin group (baseline, 7.9 mmol/L) and by 0.2 ± 0.3 mmol/L (*P* = .24) in the placebo group (baseline, 7.5 ± 0.2 mmol/L) over the study period. The between-group difference in change from baseline was significant (*P* = .018). The time course of change is shown in Figure 1B.

In the vildagliptin-treated group, the decrease in fasting plasma glucose positively correlated with the decrease in fasting liver triglyceride content at 3 months (r = 0.47; *P* = .02) and 6 months (r = 0.44; *P* = .03).

### Plasma insulin

Mean fasting plasma insulin decreased over the study period by 0.2 ± 0.2 mU/L (*P* = .22) in the vildagliptin group (baseline, 12.0 ± 1.6 mU/L) and by 1.4 ± 0.2 mU/L (*P* = .21) in the placebo group (baseline, 14.6 ± 2.0 mU/L). The between-group difference in change from baseline to endpoint was insignificant (*P* = .95). There was a decrease in fasting plasma insulin within the vildagliptin group by 4 months (12.0 ± 1.6 to 8.6 ± 1.3 mU/L; *P* = .04).

### Whole-body glucose and lipid oxidation

Fasting whole-body glucose oxidation rate did not differ between the vildagliptin and placebo groups either at baseline (0.99 ± 0.16 vs 0.87 ± 0.10 mg/kg/min) or at study endpoint (0.77 ± 0.19 vs 0.95 ± 0.14 mg/kg/min, respectively; *P* = .21 for the between-group difference in change from baseline). Similarly, the insulin-stimulated whole-body glucose oxidation rate did not differ between the vildagliptin and placebo groups at baseline (1.52 ± 0.21 vs 1.50 ± 0.08 mg/kg/min) or at the end of the study (1.55 ± 0.16 vs 1.65 ± 0.14 mg/kg/min, respectively; *P* = .65 for the between-group change from baseline).

The fasting whole-body lipid oxidation rate did not differ between the vildagliptin and placebo groups either at baseline (1.08 ± 0.07 vs 1.13 ± 0.05 mg/kg/min) or at study endpoint (1.13 ± 0.06 vs 1.05 ± 0.08 mg/kg/min, respectively, for the between-group difference in change from baseline). There was no significant difference in clamp lipid oxidation between both groups at baseline (0.89 ± 0.09 vs 0.86 ± 0.08 mg/kg/min) and the end of the study (0.91 ± 0.07 vs 0.94 ± 0.07 mg/kg/min).

The fasting whole-body energy expenditure did not change in the vildagliptin (1698 ± 92.5 vs 1673 ± 89.6 Kcal/d; *P* = .35) or placebo (1849 ± 75.3 vs 1729 ± 94.9 Kcal/d; *P* = .08) groups over 6 months. No change was observed in clamp whole-body energy expenditure in both the vildagliptin (1724 ± 77.6 vs 1676 ± 104.8 Kcal/d; *P* = .22) and placebo groups (1877 ± 96.9 vs 1798 ± 101 Kcal/d; *P* = .23).

### Plasma lipids

Mean fasting triglyceride decreased by 0.2 ± 0.1 mmol/L from a baseline of 1.5 ± 0.1 mmol/L (*P* = .05) in the vildagliptin group, compared to no change in the placebo group (0.0 ± 0.1 mmol/L from a baseline of 1.4 ± 0.1 mmol/L; *P* = .37). However, the between-group difference was not statistically significant (*P* = .17). Baseline fasting plasma triglyceride was similar in the type 2 diabetes and normal glucose-tolerant groups (1.5 ± 0.1 vs 1.5 ± 0.3 mmol/L; *P* = .90).

Plasma NEFA remained unchanged over the study period in the vildagliptin (0.48 ± 0.04 to 0.50 ± 0.05 mmol/L; *P* > .05) and placebo (0.50 ± 0.03 to 0.49 ± 0.03 mmol/L; *P* > .05) groups. Plasma HDL remained unchanged over the study period in the vildagliptin (1.41 ± 0.1 to 1.43 ± 0.1 mmol/L; *P* > .05) and placebo (1.30 ± 0.1 to 1.30 ± 0.1 mmol/L; *P* > .05) groups. Similarly, plasma very low-density lipoprotein was unchanged in the vildagliptin (0.5 ± 0.1 to 0.4 ± 0.0 mmol/L; *P* > .05) and placebo (0.5 ± 0.1 to 0.4 ± 0.0 mmol/L; *P* > .05) groups.

### Weight and percentage body fat

**Figure 3.**
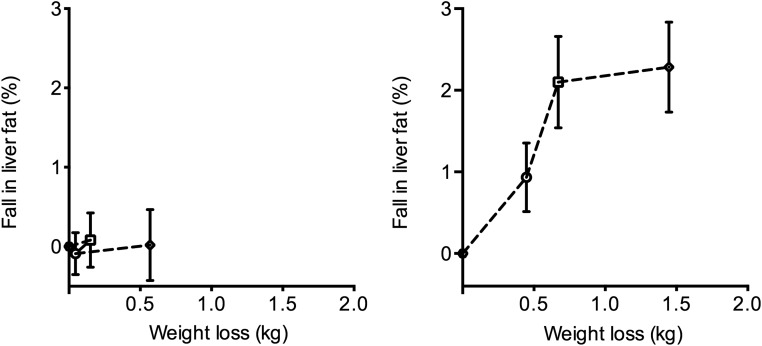
Lack of relationship between change in liver fat and change in weight for the placebo group (left panel) and the vildagliptin group (right panel). Weight loss of 0.5 kg brought about no change in liver fat in the placebo group, whereas the continuing steady weight loss in the vildagliptin group brought about no additional change in liver fat from 3 to 6 months.

Mean body weight decreased by 1.6 ± 0.5 kg from a baseline of 82.6 ± 3.4 kg (*P* = .002) in the vildagliptin group and by 0.4 ± 0.5 kg from a baseline of 92.1 ± 2.5 kg (*P* = .41) in the placebo group over the study period. The between-group difference did not reach statistical significance (*P* = .08). Modest weight loss in the placebo group was associated with no change in liver fat, and the time courses of change in weight and change in liver fat were not congruent in either group (Figure 3 and Supplemental Figure 3).

Similarly, mean percentage body fat fell over the same time period (31.1 ± 1.8 to 26.3 ± 2.2%; *P* = .003) in the vildagliptin group, with no change in the placebo group (31.4 ± 1.8 to 30.0 ± 2.1%; *P* = .08).

### Safety and tolerability

Vildagliptin was well tolerated, and there were no meaningful differences between the vildagliptin and placebo groups in the overall adverse events (AEs) profile. AEs occurred in 15 patients (68.2%) in the vildagliptin group vs 16 patients (72.7%) in the placebo group. Discontinuation due to AEs happened in one vs three patients, respectively. Serious AEs (two vs one patient, respectively) were similar between the treatment groups with no deaths. Most frequent AEs in the categories of infection and infestation (four vs five patients) and musculoskeletal and connective tissue disorders (four patients in each group). Mild hypoglycemia (<3.1 mmol/L) was reported in one patient in the vildagliptin group, and none were reported in the placebo group.

## Discussion

This study demonstrates for the first time that DPP-4 inhibition can bring about clinically useful decreases in liver triglyceride levels associated with a fall in both plasma ALT and plasma glucose. Although the patient group was selected to have well-controlled type 2 diabetes and the change in plasma glucose levels was expected to be modest, HbA_1c_ fell from an average of 6.5 to 6.0% in the group receiving vildagliptin. However, there was no change in insulin sensitivity as assessed by the euglycemic (5.5 mmol/L) hyperinsulinemic (40 mU insulin min^−2^min^−1^) clamp.

The mechanism underlying both the discovery and development of DPP-4 inhibitors was the GLP-1-mediated enhancement of pancreatic islet function (1–3). The GLP-1-mediated action brings about increased sensitivity of both α- and β-cells to glucose. However, vildagliptin has been observed to cause changes that suggest additional metabolic effects. Specifically, the rate of appearance of palmitate (ie, lipolysis) was found to be decreased, and lipid oxidation was also decreased during a euglycemic clamp study (15). During fasting, almost 80% of triglyceride in the very low-density lipoprotein secreted from the liver derives from adipose tissue (16). The rate of de novo lipogenesis rises sharply with increasing extent of liver fat accumulation and accounts for over a 3-fold greater contribution to plasma triglyceride in subjects with nonalcoholic fatty liver disease (17). It was therefore important to quantify the effect of DPP-4 inhibition upon ectopic fat in liver because this organ is central to lipoprotein homeostasis and whole-body lipid metabolism.

The present study has demonstrated a substantial fall in fasting liver triglyceride concentration during vildagliptin treatment, with levels becoming similar to that of the nondiabetic control group. The major influence on liver triglyceride is body weight, and weight and percentage body fat decreased to only a modest degree on active treatment. Figure 3 illustrates the lack of a temporal relationship between change in body weight and change in liver fat. Additionally, by applying the observation of Tiikkainen et al (18) that a 1-kg loss of body weight at steady state is associated with a decrease in liver fat of approximately 0.58%, the highly statistically significant fall in liver fat on vildagliptin therapy remained (1 mo, *P* < .01; 3 mo, *P* < .01; 6 mo, *P* < .001). The extent of the fall in liver triglyceride was clinically significant, as reflected by the reduction in plasma ALT and also by the correlations between the decreases in liver triglyceride and those of both plasma ALT and fasting plasma glucose. The improvement in liver triglyceride was less than that reported for thiazolidinedione therapy (19, 20). The mechanism underlying the fall in liver triglyceride on vildagliptin therapy must be considered. Either fat oxidation must have increased or the balance between net hepatic storage and export must have changed. The most likely explanation is a substantial decrease in storage due to the previously observed decrease in fasting lipolysis and fatty acid supply to the liver (8). De novo lipogenesis was not assessed in this study, but overall a modest increase in this process would account for the lower plasma glucose levels in the face of no decrease in HGP and would still be consistent with the observed change in hepatic triglyceride concentration. No change in whole-body lipid oxidation was observed. Hepatic export of lipid is unlikely to have increased given the 13% fall in plasma triglyceride concentration and given the results from a previous study that showed no effect of vildagliptin treatment on postprandial triglyceride levels secondary to reduced flux of lipoproteins from the liver (6).

It is known that the GLP-1 receptor is expressed on human hepatocytes (21), and a direct effect of increased GLP-1 levels is feasible. GLP-1 agonist therapy is known to decrease hepatic triglyceride levels in ob/ob mice (22), and DPP-4 inhibitors have been shown to reduce liver fat in obese mice models (23). Furthermore, DPP-4 inhibition prevents fatty liver developing during overfeeding of both wild-type and glucokinase-deficient mice (24). In the latter study, it was also demonstrated that fatty acid synthase expression was decreased. However, caution is required in drawing mechanistic information from studies on rodents that, unlike humans, are able to synthesize fatty acids from glucose in adipocytes (25)

Overall, blood glucose control improved on vildagliptin, as reflected by HbA_1c_ falling into the nondiabetic range and fasting plasma glucose falling by 0.9 mmol/L. These results are particularly noteworthy given the very low baseline HbA_1c_ of patients included in the study (mean, 6.4%) because oral hypoglycemic agents in general have effects directly proportional to initial blood glucose levels. Mean HbA_1c_ was normalized after 24 weeks of treatment with vildagliptin 50 mg bid (twice a day). Of note, the vildagliptin treatment effect on HbA_1c_ seen in this study is very robust when compared to previous data (26). In the present study, vildagliptin was added to stable metformin therapy, and it is possible that this is relevant to the extent of normalization of blood glucose levels. However, improvement in glucose control itself is unlikely to have had a direct effect upon liver fat levels, as shown by observation of no change in liver fat levels during metformin or sulfonylurea therapy (27, 28).

Basal HGP was not changed by vildagliptin treatment, and this confirms previous observations during both vildagliptin and sitagliptin therapy (15, 29). In contrast, in a single-dose, postmeal, and overnight study (30), there was on average 0.3 mg/kg/min less HGP in the vildagliptin group relative to the placebo group from the beginning of the dinner meal until the next morning. The lack of improvement in fasting HGP despite a fall in liver fat must be considered. This may relate to the very well-controlled type 2 diabetes under study because HGP in diabetes has been observed to decline to nondiabetic levels below a plasma glucose of approximately 8 mmol/L (31). Further work is required to evaluate the effect of vildagliptin on HGP during a low, physiological increase in insulin concentrations such as occurs after meals, especially because an increase in suppression of HGP under these circumstances would be predicted by the decrease in hepatic triglyceride. It must be noted that the clamp insulin levels were optimal to measure peripheral rather than hepatic insulin sensitivity.

Treatment with vildagliptin added to metformin over 6 months resulted in a 1.6-kg weight loss vs baseline. While overall treatment with DPP-4 inhibitors is weight neutral, it was previously reported (26) that from a similarly low baseline level of glycemia, as in the present study (baseline mean HbA_1c_, 6.6%), vildagliptin therapy reduced body weight by 1.1 kg over a 2-year period (32). Furthermore, examination of weight as a function of fasting plasma glucose in a pooled analysis of > 2000 patients demonstrated that a negative calorie balance occurred in those with glucose levels below the renal threshold (32).

Limitations of the present study must be considered. Examination of whole-body insulin sensitivity by conducting euglycemic hyperinsulinemic clamps at a range of insulin concentrations would have been ideal. Similarly, detailed examination of hepatic insulin sensitivity within the physiological range of insulin concentrations would be of interest in relation to the substantial decrease in hepatic triglyceride. Finally, because the starting mean hepatic triglyceride level in the vildagliptin group was by random chance higher than that of the placebo group, it is important to be sure that the observed change was not merely a tendency to the mean. To exclude this possibility, when subjects were pair-matched between the groups for baseline liver triglyceride level with values within 0.2%, 12 pairs were identified, and the vildagliptin-treated levels fell over the 6-month period (6.29 ± 1.01 to 4.06 ± 0.61; *P* < .02), whereas there was no change on placebo (6.35 ± 1.03 to 6.57 ± 1.57; *P* = .73).

In summary, this study has demonstrated that a clinically significant change in hepatic triglyceride levels is achieved on vildagliptin therapy and that this is unrelated to the small change in body weight.
